# Supplementary material for: College from home during COVID-19: A mixed-methods study of heterogeneous experiences
Source: PLoS One. 2021 Jun 28;16(6):e0251580. doi: 10.1371/journal.pone.0251580 (PMC8238179; doi:10.1371/journal.pone.0251580)
Supplement: S1 Table — (DOCX) [file pone.0251580.s001.docx]

##### **S1 Table. Participant Demographics.**

|  | 2019^3^  n=253  n (%) | 2020^3^  n=147  n (%) | Interviewees  n=27  n (%) |
| --- | --- | --- | --- |
| **Gender** |  |  |  |
| Women | 126 (50) | 81 (55) | 16 (59) |
| Nonbinary | 2 (1) | 2 (1) | 0 |
| Men | 125 (49) | 64 (44) | 11 (41) |
| **Race/Ethnicity** |  |  |  |
| Asian or Asian-American | 123 (49) | 83 (56) | 19 (70) |
| Minority^1^ or Biracial | 51 (20) | 21 (14) | 3 (11) |
| White | 79 (31) | 42 (29) | 5 (19) |
| Not provided | 0 | 1 (1) | 0 |
| **Low SES^2^** | N/A | 38 (26) | 10 (37) |
| **Year in College** |  |  |  |
| First-year | 199 (79) | 37 (25) | 9 (33) |
| Second-year | 54 (21) | 63 (43) | 7 (26) |
| Third-year | 0 | 43 (29) | 11 (41) |
| Fourth-year | 0 | 4 (3) | 0 |

*Notes:* No significant group differences.

^1^Includes African or African American, Alaska Native, Native American, Latinx, Pacific Islander

^2^SES Measure not included on 2019 survey

^3^ 90 participants took part in both 2019 and 2020 phases
