# Supplementary material for: College from home during COVID-19: A mixed-methods study of heterogeneous experiences
Source: PLoS One. 2021 Jun 28;16(6):e0251580. doi: 10.1371/journal.pone.0251580 (PMC8238179; doi:10.1371/journal.pone.0251580)
Supplement: S2 Table — (DOCX) [file pone.0251580.s002.docx]

**S2 Table. 2019 and 2020 ESM methods and compliance.**

|  | 2019 full sample (n = 253) | 2019 pooled sample (n = 90) | 2020 full sample (n = 147) | 2020 pooled sample (n = 90) |
| --- | --- | --- | --- | --- |
| Time delivered | 10:00 AM | 10:00 AM | 10:00 AM | 10:00 AM |
| Hours to complete | 10 | 10 | 12 | 12 |
| Average time of day completed | 12:27 PM | 12:22 PM | 12:21 PM | 12:20 PM |
| Average duration | 6m 8s | 7m 21s | 7m 22s | 6m 17s |
| Compliance | 91.72% | 95.32% | 90.35% | 95.37% |
| Number of surveys/person | 1 -14 | 10 - 14 | 1 - 18 | 9 - 18 |
