# Supplementary material for: College from home during COVID-19: A mixed-methods study of heterogeneous experiences
Source: PLoS One. 2021 Jun 28;16(6):e0251580. doi: 10.1371/journal.pone.0251580 (PMC8238179; doi:10.1371/journal.pone.0251580)
Supplement: S3 Table — (DOCX) [file pone.0251580.s003.docx]

**S3 Table. 2019 and 2020 comparisons.**

| Variable | 2019 | | 2020 | | *F* (df = 1, 398) | *p* |
| --- | --- | --- | --- | --- | --- | --- |
|  | *M* | *SD* | *M* | *SD* |  |  |
| Depressive Symptoms | 12.21 | 8.59 | 12.42 | 9.08 | 0.05 | .82 |
| Anxiety | 45.85 | 10.70 | 44.36 | 10.06 | 1.89 | .17 |
| Stress | 27.66 | 7.54 | 26.94 | 6.85 | 0.91 | .34 |
| Loneliness | 22.70 | 5.59 | 22.22 | 5.10 | 0.72 | .40 |
