# Supplementary material for: College from home during COVID-19: A mixed-methods study of heterogeneous experiences
Source: PLoS One. 2021 Jun 28;16(6):e0251580. doi: 10.1371/journal.pone.0251580 (PMC8238179; doi:10.1371/journal.pone.0251580)
Supplement: S4 Table — (DOCX) [file pone.0251580.s004.docx]

**S4 Table. Time 1 and Time 2 (2020) comparisons.**

| Variable | Time 1 | | Time 2 | | Δ Within person | *F* (df = 1, 141) | *p* |
| --- | --- | --- | --- | --- | --- | --- | --- |
|  | *M* | *SD* | *M* | *SD* |  |  |  |
| Depressive Symptoms | 12.42 | 9.08 | 13.51 | 9.40 | 1.09 | 3.81 | .053 |
| Anxiety | 44.36 | 10.06 | 45.29 | 10.87 | 0.93 | 2.79 | .10 |
| Stress | 26.94 | 6.85 | 26.63 | 7.77 | -0.31 | 0.15 | .70 |
| Loneliness | 22.22 | 5.10 | 22.18 | 4.99 | -0.04 | 0.36 | .55 |
