# Supplementary material for: College from home during COVID-19: A mixed-methods study of heterogeneous experiences
Source: PLoS One. 2021 Jun 28;16(6):e0251580. doi: 10.1371/journal.pone.0251580 (PMC8238179; doi:10.1371/journal.pone.0251580)
Supplement: S5 Table — (DOCX) [file pone.0251580.s005.docx]

**S5 Table. Differences in 2020 ESM reports over time.**

| Variable | b | *t* (df = 2259) | *p* |
| --- | --- | --- | --- |
| Depressive Symptoms | 0.07 | 4.57 | < .001 |
| Stress | 0.04 | 2.80 | .005 |
| Depressed Affect | 0.02 | 4.86 | < .001 |
| Anxious | 0.01 | 2.94 | .003 |
| Lonely | 0.00 | 0.44 | .66 |
| Composite Negative Affect | 0.02 | 5.83 | < .001 |

*Notes:* Levenne’s Test suggests significant heterogeneity of the residuals for each of the variables.
