# Supplementary material for: College from home during COVID-19: A mixed-methods study of heterogeneous experiences
Source: PLoS One. 2021 Jun 28;16(6):e0251580. doi: 10.1371/journal.pone.0251580 (PMC8238179; doi:10.1371/journal.pone.0251580)
Supplement: S6 Table — (DOCX) [file pone.0251580.s006.docx]

**S6 Table. Differences in pooled ESM reports based on Time 1 Depression Scores.**

| Variable | *𝛃* | *t* | df | *p* |
| --- | --- | --- | --- | --- |
| Depressive Symptoms^a^ | 0.40 | 10.97 | 1299 | < .001 |
| Stress | 0.29 | 7.89 | 1262 | < .001 |
| Depressed Affect^b^ | 0.32 | 10.31 | 2655 | < .001 |
| Anxious Affect | 0.17 | 5.41 | 2655 | <.001 |
| Loneliness | 0.26 | 7.75 | 2655 | <.001 |
| Composite Negative Affect | 0.34 | 10.76 | 2655 | <.001 |

*Notes:* a. Measured weekly via PHQ-4 b. Measured twice per week via single-item
