# Supplementary material for: College from home during COVID-19: A mixed-methods study of heterogeneous experiences
Source: PLoS One. 2021 Jun 28;16(6):e0251580. doi: 10.1371/journal.pone.0251580 (PMC8238179; doi:10.1371/journal.pone.0251580)
Supplement: S7 Table — (DOCX) [file pone.0251580.s007.docx]

**S7 Table. Differences in pooled ESM reports based on Time 1 Anxiety Scores.**

| Variable | *𝛃* | *t* | df | *p* |
| --- | --- | --- | --- | --- |
| Depressive Symptoms^a^ | 0.43 | 11.80 | 1299 | < .001 |
| Stress | 0.41 | 11.12 | 1262 | < .001 |
| Depressed Affect^b^ | 0.37 | 11.34 | 2655 | < .001 |
| Anxious Affect | 0.19 | 5.74 | 2655 | <.001 |
| Loneliness | 0.26 | 7.58 | 2655 | <.001 |
| Composite Negative Affect | 0.50 | 15.20 | 2655 | <.001 |

*Notes:* a. Measured weekly via PHQ-4 b. Measured twice per week via single-item
