# Supplementary material for: College from home during COVID-19: A mixed-methods study of heterogeneous experiences
Source: PLoS One. 2021 Jun 28;16(6):e0251580. doi: 10.1371/journal.pone.0251580 (PMC8238179; doi:10.1371/journal.pone.0251580)
Supplement: S8 Table — (DOCX) [file pone.0251580.s008.docx]

**S8 Table. Differences in pooled ESM reports based on Time 1 Loneliness Scores.**

| Variable | *𝛃* | *t* | df | *p* |
| --- | --- | --- | --- | --- |
| Depressive Symptoms^a^ | 0.25 | 7.15 | 1299 | < .001 |
| Stress | 0.19 | 5.44 | 1262 | < .001 |
| Depressed Affect^b^ | 0.14 | 4.78 | 2655 | < .001 |
| Anxious Affect | 0.05 | 1.74 | 2655 | .08 |
| Loneliness | 0.15 | 4.86 | 2655 | <.001 |
| Composite Negative Affect | 0.23 | 7.62 | 2655 | <.001 |

*Notes:* a. Measured weekly via PHQ-4 b. Measured twice per week via single-item
