# Supplementary material for: College from home during COVID-19: A mixed-methods study of heterogeneous experiences
Source: PLoS One. 2021 Jun 28;16(6):e0251580. doi: 10.1371/journal.pone.0251580 (PMC8238179; doi:10.1371/journal.pone.0251580)
Supplement: S9 Table — (DOCX) [file pone.0251580.s009.docx]

**S9 Table. Multi-level models of Year, Day, and Time 1 Depression interactions.**

| Depressive Symptoms Stress | | | | | | | | | |  |
| --- | --- | --- | --- | --- | --- | --- | --- | --- | --- | --- |
|  | 𝛃 | 95% CI | *t* (df) | p-value | 𝛃 | 95% CI | | *t* (df) | | p-value |
| Intercept | -0.13 | -0.39 – 0.12 | -1.04(1293) | .30 | 0.12 | -0.13 – -0.37 | 0.96 (1256) | | .34 | |
| Day | 0.01 | -0.02 – 0.04 | 0.67 (1293) | .50 | -0.00 | -0.03 – 0.03 | -0.12 (1256) | | .91 | |
| Time 1 Dep | 0.69 | 0.44 – 0.95 | 5.45 (1293) | <.001* | 0.37 | 0.13 – 0.61 | 3.02 (1256) | | .003* | |
| Year | -0.01 | -0.15 – 0.13 | -0.12 (1293) | .91 | -0.09 | -0.23 – 0.05 | -1.31 (1256) | | .19 | |
| Year × Day | 0.01 | -0.01 – 0.02 | 0.81 (1293) | .42 | 0.01 | -0.01 – 0.02 | 0.807 (1256) | | .43 | |
| Year × Time 1 Dep | -0.14 | -0.28 – 0.01 | -1.81 (1293) | .07 | 0.00 | -0.14 - 0.14 | 0.01 (1256) | | .99 | |
| Day × Time 1 Dep | -0.04 | -0.06 – -0.01 | -2.49 (1293) | .01* | -0.03 | -0.06 - 0.00 | -1.87 (1256) | | .06 | |
| Year × Day × Time 1 Dep | 0.01 | -0.00 – 0.03 | 1.74 (1293) | .08 | 0.01 | -0.01 - 0.03 | 1.23 (1256) | | .22 | |
| Depressed affect Anxiety | | | | | | | | | | |
|  | 𝛃 | 95% CI | *t* (df) | p-value | 𝛃 | 95% CI | *t* (df) | | p-value | |
| Intercept | -0.01 | -0.22 - 0.21 | -0.06 (2649) | .95 | 0.21 | -0.01 - 0.44 | 1.90 (2649) | | .06 | |
| Day | -0.01 | -0.03 - 0.02 | -0.53 (2649) | .60 | -0.03 | -0.05 - -0.00 | -2.27 (2649) | | .02* | |
| Time 1 Dep | 0.89 | 0.67 - 1.10 | 8.08 (2649) | <.001* | 0.13 | -0.08 - 0.35 | 1.21 (2649) | | .23 | |
| Year | -0.09 | -0.21 - 0.03 | -1.46 (2649) | .14 | -0.16 | -0.28 - -0.04 | -2.65 (2649) | | .01* | |
| Year × Day | 0.01 | 0.00 - 0.03 | 2.08 (2649) | .04☨ | 0.02 | 0.01 - 0.03 | 3.02 (2649) | | .003* | |
| Year × Time 1 Dep | -0.30 | -0.43 - -0.18 | -4.71 (2649) | <.001* | 0.02 | -0.10 - 0.15 | 0.38 (2649) | | .70 | |
| Day × Time 1 Dep | -0.07 | -0.10 - 0.05 | -5.84 (2649) | <.001* | -0.01 | -0.03 - 0.02 | -0.54 (2649) | | .59 | |
| Year × Day × Time 1 Dep | 0.04 | 0.02 - 0.05 | 5.40 (2649) | <.001* | 0.01 | -0.01 - 0.02 | 0.74 (2649) | | .46 | |
| \|  \| Loneliness Composite Negative Affect \| \| \| \| \| \| \| \| \| \| \| --- \| --- \| --- \| --- \| --- \| --- \| --- \| --- \| --- \| --- \| --- \| \|  \| \| 𝛃 \| 95% CI \| *t* (df) \| p-value \| 𝛃 \| 95% CI \| *t* (df) \| p-value \| \| Intercept \| \| -0.41 \| -0.64 - -0.73 \| -3.38 (2649) \| .001* \| 1.19 \| 0.99 - 1.40 \| 11.53 (2649) \| <.001* \| \| Day \| \| 0.01 \| -0.02 - 0.03 \| 0.59 (2649) \| .56 \| 0.01 \| -0.01 - 0.04 \| 1.23 (2649) \| 0.22 \| \| Time 1 Dep \| \| 0.62 \| 0.40 - 0.84 \| 5.55 (2649) \| <.001* \| 0.98 \| 0.78 - 1.18 \| 9.54 (2649) \| <.001* \| \| Year \| \| 0.54 \| 0.13 - 0.37 \| 4.01 (2649) \| <.001* \| -0.83 \| -0.94 - -0.71 \| -14.21 (2649) \| <.001* \| \| Year × Day \| \| -0.00 \| -0.02 - 0.01 \| -0.51 (2649) \| .61 \| -0.00 \| -0.02 - 0.01 \| -0.44 (2649) \| .66 \| \| Year × Time 1 Dep \| \| -0.16 \| -0.29 - -0.03 \| -2.47 (2649) \| .01* \| -0.44 \| -0.56 - -0.32 \| -7.32 (2649) \| <.001* \| \| Day × Time 1 Dep \| \| -0.04 \| -0.07 - 0.02 \| -3.40 (2649) \| .001* \| -0.02 \| -0.04 - 0.00 \| -1.70 (2649) \| .09 \| \| Year × Day × Time 1 Dep \| \| 0.02 \| 0.01 - 0.03 \| 2.69 (2649) \| .01* \| 0.01 \| -0.00 - 0.02 \| 1.63 (2649) \| .10 \| | | | | | | | | | | |

***Notes:*** ☨ = Non-significant after applying Benjamini-Hochberg procedure ×
