# Supplementary material for: College from home during COVID-19: A mixed-methods study of heterogeneous experiences
Source: PLoS One. 2021 Jun 28;16(6):e0251580. doi: 10.1371/journal.pone.0251580 (PMC8238179; doi:10.1371/journal.pone.0251580)
Supplement: S10 Table — (DOCX) [file pone.0251580.s010.docx]

**S10 Table. Multi-level models of Year, Day, and Time 1 Anxiety interactions.**

| Depressive Symptoms Stress | | | | | | | | | |  |
| --- | --- | --- | --- | --- | --- | --- | --- | --- | --- | --- |
|  | 𝛃 | 95% CI | *t* (df) | p-value | 𝛃 | 95% CI | | *t* (df) | | p-value |
| Intercept | -0.23 | -0.48 - 0.03 | -1.76 (1293) | .08 | -0.00 | -0.25 - 0.24 | -0.01 (1256) | | .99 | |
| Day | 0.01 | -0.02 - 0.04 | 0.58 (1293) | .56 | 0.00 | -0.03 - 0.03 | 0.11 (1256) | | .91 | |
| Time 1 Anx | 0.60 | 0.36 - 0.84 | 4.83 (1293) | <.001* | 0.44 | 0.22 - 0.67 | 3.83 (1256) | | <.001* | |
| Year | 0.05 | -0.09 - 0.19 | 0.69 (1293) | .49 | -0.02 | -0.15 - 0.12 | -0.23 (1256) | | .82 | |
| Year × Day | 0.01 | -0.01 - 0.02 | 0.94 (1293) | .35 | 0.00 | -0.01 - 0.02 | 0.59 (1256) | | .55 | |
| Year × Time 1 Anx | -0.09 | -0.24 - 0.06 | -1.20 (1293) | .23 | 0.02 | -0.12 - 0.16 | 0.34 (1256) | | .74 | |
| Day × Time 1 Anx | -0.02 | -0.05 - 0.01 | -1.43 (1293) | .15 | -0.02 | -0.05 - 0.01 | -1.37 (1256) | | .17 | |
| Year × Day × Time 1 Anx | 0.01 | -0.00 - 0.03 | 1.60 (1293) | .11 | 0.01 | -0.01 - 0.02 | 0.88 (1256) | | .38 | |
| Depressed affect Anxiety | | | | | | | | | | |
|  | 𝛃 | 95% CI | *t* (df) | p-value | 𝛃 | 95% CI | *t* (df) | | p-value | |
| Intercept | -0.10 | -0.32 - 0.12 | -0.93 (2649) | .35 | 0.17 | -0.05 - 0.39 | 1.52 (2649) | | .13 | |
| Day | -0.01 | -0.03 - 0.02 | -0.41 (2649) | .68 | -0.03 | -0.05 - -0.00 | -2.07 (2649) | | .03☨ | |
| Time 1 Anx | 0.66 | 0.46 - 0.87 | 6.31 (2649) | <.001* | 0.10 | -0.11 - 0.30 | 0.95 (2649) | | .35 | |
| Year | -0.03 | -0.15 - 0.09 | -0.51 (2649) | .61 | -0.13 | -0.25 - -0.01 | -2.11 (2649) | | .03☨ | |
| Year × Day | 0.01 | -0.00 - 0.03 | 2.04 (2649) | .04☨ | 0.02 | 0.01 - 0.03 | 2.91 (2649) | | .003* | |
| Year × Time 1 Anx | -0.16 | -0.28 - -0.03 | -2.50 (2649) | .01* | 0.07 | -0.06 - 0.20 | 1.13 (2649) | | .27 | |
| Day × Time 1 Anx | -0.05 | -0.07 - -0.02 | -3.88 (2649) | <.001* | 0.00 | -0.02 - 0.02 | 0.09 (2649) | | .97 | |
| Year × Day × Time 1 Anx | 0.03 | 0.01 - 0.04 | 4.03 (2649) | <.001* | 0.00 | -0.01 - 0.01 | 0.01 (2649) | | .95 | |
| \|  \| Loneliness Composite Negative Affect \| \| \| \| \| \| \| \| \| \| \| --- \| --- \| --- \| --- \| --- \| --- \| --- \| --- \| --- \| --- \| --- \| \|  \| \| 𝛃 \| 95% CI \| *t* (df) \| p-value \| 𝛃 \| 95% CI \| *t* (df) \| p-value \| \| Intercept \| \| -0.50 \| -0.73 - -0.26 \| -4.10 (2649) \| <.001* \| 1.12 \| 0.92 - 1.33 \| 10.68 (2649) \| <.001* \| \| Day \| \| 0.01 \| -0.02 - 0.03 \| 0.72 (2649) \| .47 \| 0.02 \| -0.01 - 0.04 \| 1.09 (2649) \| .28 \| \| Time 1 Anx \| \| 0.51 \| 0.30 - 0.72 \| 4.77 (2649) \| <.001* \| 0.7 \| 0.57 - 0.96 \| 7.72 (2649) \| <.001* \| \| Year \| \| 0.30 \| 0.18 - 0.42 \| 4.87 (2649)) \| <.001* \| -0.79 \| -0.90 - -0.67 \| -13.39 (2649) \| <.001* \| \| Year × Day \| \| -0.00 \| -0.02 - 0.01 \| -0.65 (2649) \| .52 \| -0.01 \| -0.02 - 0.01 \| -0.32 (2649) \| .75 \| \| Year × Time 1 Anx \| \| -0.09 \| -0.21 - 0.045 \| -1.33 (2649) \| .18 \| -0.32 \| -0.44 - -0.20 \| -5.32 (2649) \| <.001* \| \| Day × Time 1 Anx \| \| -0.02 \| -0.04 - 0.01 \| -1.26 (2649) \| .21 \| 0.00 \| 0.02 - 0.03 \| 0.26 (2649) \| .80 \| \| Year × Day × Time 1 Anx \| \| 0.01 \| -0.01 - 0.02 \| 0.74 (2649) \| .46 \| 0.00 \| -0.01 - 0.01 \| -0.08 (2649) \| .93 \| | | | | | | | | | | |

***Notes:*** ☨ = Non-significant after applying Benjamini-Hochberg procedure
