# Supplementary material for: College from home during COVID-19: A mixed-methods study of heterogeneous experiences
Source: PLoS One. 2021 Jun 28;16(6):e0251580. doi: 10.1371/journal.pone.0251580 (PMC8238179; doi:10.1371/journal.pone.0251580)
Supplement: S11 Table — (DOCX) [file pone.0251580.s011.docx]

**S11 Table. Multi-level models of Year, Day, and Time 1 Loneliness interactions.**

| Depressive Symptoms Stress | | | | | | | | | |  |
| --- | --- | --- | --- | --- | --- | --- | --- | --- | --- | --- |
|  | 𝛃 | 95% CI | *t* (df) | p-value | 𝛃 | 95% CI | | *t* (df) | | p-value |
| Intercept | -0.12 | -0.39 - 0.14 | -0.90 (1293) | .37 | 0.12 | -0.14 - 0.38 | 0.94 (1256) | | .35 | |
| Day | 0.01 | -0.02 - 0.04 | 0.67 (1293) | .51 | -0.00 | -0.03 - 0.03 | -0.05 (1256) | | .96 | |
| Time 1 Lonely | 0.60 | 0.36 - 0.85 | 4.83 (1293) | <.001* | 0.21 | 0.08 - 0.54 | 2.68 (1256) | | .01* | |
| Year | -0.01 | -0.16 - 0.13 | -0.20 (1293) | .84 | -0.09 | -0.23 - 0.04 | -1.33 (1256) | | .18 | |
| Year × Day | 0.01 | -0.01 - 0.02 | 0.83 (1293) | .41 | 0.01 | -0.01 - 0.02 | 0.75 (1256) | | .45 | |
| Year × Time 1 Lonely | -0.21 | -0.36 - -0.06 | -2.80 (1293) | .01* | -0.05 | -0.19 - 0.09 | -0.71 (1256) | | .48 | |
| Day × Time 1 Lonely | -0.04 | -0.07 - -0.02 | -3.05 (1293) | .002* | -0.03 | -0.06 - -0.01 | -2.33 (1256) | | .02* | |
| Year × Day × Time 1 Lonely | 0.03 | 0.01 -0.04 | 3.13 (1293) | .002* | 0.02 | 0.00 - 0.03 | 2.18 (1256) | | .03☨ | |
| Depressed affect Anxiety | | | | | | | | | | |
|  | 𝛃 | 95% CI | *t* (df) | p-value | 𝛃 | 95% CI | *t* (df) | | p-value | |
| Intercept | 0.01 | -0.22 - 0.24 | 0.09 (2649) | .93 | 0.22 | -0.00 - 0.45 | 1.93 (2649) | | .05 | |
| Day | -0.01 | -0.03 - 0.02 | -0.49 (2649) | .62 | -0.03 | -0.05 - -0.00 | -2.22 (2649) | | .03☨ | |
| Time 1 Lonely | 0.41 | 0.20 - 0.63 | 3.86 (2649) | <.001* | -0.15 | -0.36 - 0.05 | -1.45 (2649) | | .15 | |
| Year | -0.10 | -0.22 - 0.03 | -1.58 (2649) | .11 | -0.16 | -0.28 - 0.04 | -2.68 (2649) | | .01* | |
| Year × Day | 0.01 | 0.00 - 0.03 | 2.01 (2649) | .04☨ | 0.02 | 0.01 - 0.03 | 2.94 (2649) | | .003* | |
| Year × Time 1 Lonely | -0.15 | -0.28 - 0.02 | -2.23 (2649) | .03☨ | 0.15 | 0.03 - 0.28 | 2.38 (2649) | | .02* | |
| Day × Time 1 Lonely | -0.04 | -0.06 - 0.01 | -2.98 (2649) | .003* | 0.00 | -0.02 - 0.03 | 0.31 (2649) | | .76 | |
| Year × Day × Time 1 Lonely | 0.02 | 0.01 - 0.03 | 2.73 (2649) | .01* | -0.00 | -0.02 - 0.01 | -0.43 (2649) | | .67 | |
| \|  \| Loneliness Composite Negative Affect \| \| \| \| \| \| \| \| \| \| \| --- \| --- \| --- \| --- \| --- \| --- \| --- \| --- \| --- \| --- \| --- \| \|  \| \| 𝛃 \| 95% CI \| *t* (df) \| p-value \| 𝛃 \| 95% CI \| *t* (df) \| p-value \| \| Intercept \| \| -0.40 \| -0.64 - -0.17 \| -3.39 (2649) \| <.001* \| 1.22 \| 1.00 - 1.43 \| 11.25 (2649) \| <.001* \| \| Day \| \| 0.01 \| -0.02 - 0.03 \| 0.67 (2649) \| .51 \| 0.01 \| -0.01 - 0.04 \| 1.06 (2649) \| .29 \| \| Time 1 Lonely \| \| 0.64 \| 0.43 - 0.85 \| 6.06 (2649) \| <.001* \| 0.51 \| 0.32 - 0.71 \| 5.10 (2649) \| <.001* \| \| Year \| \| 0.24 \| 0.12 - 0.37 \| 3.97 (2649) \| <.001* \| -0.84 \| -0.96 - -0.72 \| -14.13 (2649) \| <.001* \| \| Year × Day \| \| 0.00 \| -0.02 - 0.01 \| -0.61 (2649) \| .54 \| 0.00 \| -0.02 - 0.01 \| -0.34 (2649) \| .73 \| \| Year × Time 1 Lonely \| \| -0.27 \| -0.40 - -0.14 \| -4.16 (2649) \| <.001* \| -0.24 \| -0.37 - -0.12 \| -3.94 (2649) \| <.001* \| \| Day × Time 1 Lonely \| \| -0.04 \| -0.06 - 0.01 \| -2.88 (2649) \| <.001* \| 0.00 \| -0.02 - 0.02 \| 0.17 (2649) \| .87 \| \| Year × Day × Time 1 Lonely \| \| 0.01 \| 0.00 0.03 \| 2.08 (2649) \| .04☨ \| 0.00 \| -0.01 - 0.01 \| -0.25 (2649) \| .81 \| | | | | | | | | | | |

***Notes:*** ☨ = Non-significant after applying Benjamini-Hochberg procedure
