# Supplementary material for: College from home during COVID-19: A mixed-methods study of heterogeneous experiences
Source: PLoS One. 2021 Jun 28;16(6):e0251580. doi: 10.1371/journal.pone.0251580 (PMC8238179; doi:10.1371/journal.pone.0251580)
Supplement: Attachment — Submitted filename: WN review of MS.docx [file pone.0251580.s012.docx]

WN review of PONE-20-34654, “College from home during COVID-10: A mixed methods study of heterogeneous experiences”

**Review comments for the authors**

The study uses an interesting methodology to sample data in snapshots collected regularly via text message, so data gathered at regular intervals across a couple of years. That the study has been under way since before the pandemic provided an opportunity to gather data on students’ experiences. There is obviously a very rich data set here.

I have a concern about Time2 (March 2020) as the key point for comparison. This was very early in the pandemic, particularly in the US, where lockdowns and other measures were only just beginning. A justification for this early view would be helpful as motivation for this study. The earliest comparisons suggest that some students were less-stressed in March 2020 compared to the earlier comparison points. Could this be a novelty effect? Was this somehow accounted for? Does the claim about low-stress hold up given the earliness of the data gathering? The links to coping strategies are important for us as educators to consider how to best support students through difficult circumstances.

I note that, for international readers of this paper, ‘spring’ and ‘autumn’ reference Northern Hemisphere seasons and this confusion could be obviated by using months instead of seasonal references. It would also be helpful to include a statement about how the local area and university were impacted and by what timeline as the pandemic grew and public health measures came into force. What were these? How did your university respond? This local context is important for considering the results and implications of this study. Further, do students take year-long courses at your university? Or all of them semester-long? Or a combination? If year-long, students may have had time to develop relationships, but if semester-long, the absence of personal interactions may be more acutely felt.

This study is embedded in a larger one, which gives a good grounding for asking the questions in this study. The analyses provide reasonable means to answering them. The statistical tools offer fine-grained and intensive data about the effects on and experiences for undergraduate students through the pandemic. The statistical analysis in the earlier section is detailed and reasonable, and the two sets of framing offer reasonable grounds to interpret the stats through the interviews.

I think limitations for aggregating big data needs to be acknowledged. There were also differences between Likert Scale ratings, and thus these would be interpreted differently. Similarly, self-report data is often called into question. How were these issues dealt with when the results were interrogated?

A growing literature base demonstrates and details challenges undergraduates faced during 2020/covid. Some of these are local-level challenges faced by students and the value of this study is the fine-grained detail that highlights and shows comparisons with earlier years. The study reports themes/challenges that may be common with what other research has reported, but the depth present here adds to this literature with its fine-grained detail.

I’d like to know more about why authors chose to organize themes from interviews as challenges and strategies, even as this is presumably a choice about how to present results.

There will be further interesting responses to the implications of the pandemic at the university level, particularly as many universities are pushing toward more online work while reorganizing/restructuring in response to financial strains.

There are important messages in the interview analysis, especially around social interactions that are afforded by on campus/in person activity that is necessarily associated with being a college student. This sort of engagement is perhaps underappreciated by those who advocate online learning for efficiency, cost etc, which is another key important result of the reported research—to draw attention to this aspect of college/university life and the social/protective factors entailed.

Where is the interview protocol?

p. 38, line 847 says there was training offered in CBT. The study didn’t talk about training.

The final discussion points do not really follow from the paper and what was presented.

There are very few minor production errors, misplaced apostrophes, etc

**Review comments for the editor**

I must admit that I have limited experience with the range of statistical tools used in this research. This limits my ability to comment in too much detail on the appropriateness of their use or the veracity of the analyses or claims generated.
